# LINC-PINT impedes DNA repair and enhances radiotherapeutic response by targeting DNA-PKcs in nasopharyngeal cancer

**DOI:** 10.1038/s41419-021-03728-2

**Published:** 2021-05-07

**Authors:** You-hong Wang, Zhen Guo, Liang An, Yong Zhou, Heng Xu, Jing Xiong, Zhao-qian Liu, Xiao-ping Chen, Hong-hao Zhou, Xiong Li, Tao Liu, Wei-hua Huang, Wei Zhang

**Affiliations:** 1 Department of Clinical Pharmacology, Xiangya Hospital, Central South University, 87 Xiangya Road, 410008 Changsha, P. R. China; 2 Institute of Clinical Pharmacology, Central South University, Hunan Key Laboratory of Pharmacogenetics, 110 Xiangya Road, 410008 Changsha, P. R. China; 3 Engineering Research Center of Applied Technology of Pharmacogenomics, Ministry of Education, 110 Xiangya Road, 410008 Changsha, P. R. China; 4 National Clinical Research Center for Geriatric Disorders, 87 Xiangya Road, 410008 Changsha, Hunan P. R. China; 5 The Third Xiangya Hospital of Central South University, Central South University, 138 Tongzipo Road, 410013 Changsha, P. R. China; 6 Department of Laboratory Medicine, National Key Laboratory of Biotherapy/ Collaborative Innovation Center of Biotherapy and Cancer Center, West China Hospital, Sichuan University, 610000 Chengdu, P. R. China; 7 Department of Gynaecology and Obstetrics, The Second Xiangya Hospital of Central South University, Central South University, 410008 Changsha, P. R. China; 8 The First Affiliated Hospital of Guangdong Pharmaceutical University, 510060 Guangzhou, P. R. China; 9 Shenzhen Center for Chronic Disease Control and Prevention, Shenzhen, 518020 Guangdong, P. R. China

**Keywords:** Cancer therapeutic resistance, DNA damage response

## Abstract

Radioresistance continues to be the leading cause of recurrence and metastasis in nasopharyngeal cancer. Long noncoding RNAs are emerging as regulators of DNA damage and radioresistance. LINC-PINT was originally identified as a tumor suppressor in various cancers. In this study, LINC-PINT was significantly downregulated in nasopharyngeal cancer tissues than in rhinitis tissues, and low LINC-PINT expressions showed poorer prognosis in patients who received radiotherapy. We further identified a functional role of LINC-PINT in inhibiting the malignant phenotypes and sensitizing cancer cells to irradiation in vitro and in vivo. Mechanistically, LINC-PINT was responsive to DNA damage, inhibiting DNA damage repair through ATM/ATR-Chk1/Chk2 signaling pathways. Moreover, LINC-PINT increased radiosensitivity by interacting with DNA-dependent protein kinase catalytic subunit (DNA-PKcs) and negatively regulated the expression and recruitment of DNA-PKcs. Therefore, these findings collectively support the possibility that LINC-PINT serves as an attractive target to overcome radioresistance in NPC.

## Introduction

Nasopharyngeal carcinoma (NPC) remains endemic in the east and southeast Asia, with 90,300 new cases estimated in 2018 (ref. ^1,2^). Since NPC cells show sensitivity to ionizing radiation (IR), radiotherapy emerges as a cornerstone for NPC patients. Compared with three-dimensional radiotherapy, Intensity-modulated Radiotherapy (IMRT) becomes a preferred option for NPC, delivering a higher survival rate with reduced toxicities^3^. However, local recurrence and distant metastasis occur in 20–30% NPC patients since intrinsic or acquired resistance developed^2^. Radioresistance causes NPC therapeutic failure. A potential method to improve outcomes is to sensitize tumor cells to irradiation. Therefore, it is needed to devote great attention to molecular mechanisms of NPC radiosensitivity.

DNA damage is the core of radiotherapy. In response to IR and other insults, cells have evolved an intricate network to sense, detect, and repair DNA damage, collectively known as the DNA damage response (DDR)^4^. Following the IR-induced double-strand breaks (DSBs), DDR coordinates cell cycle progression and DSB repair to determine cell survival or death. Thus, targeting DDR pathways may have the potential to affect the outcome of cancer treatment, sensitizing, or desensitizing cancer cells to irradiation^4,5^. However, mechanistic studies in DDR have focused on proteins, the functions of noncoding RNA remain largely unknown.

Long noncoding RNAs (lncRNAs) are defined as noncoding transcripts comprising more than 200 nt and lacking the potential to encode proteins^6^. LncRNAs are aberrantly expressed in various cancers, regulating multiple biological processes, such as cell proliferation, migration, invasion, and apoptosis^7,8^. Interestingly, some lncRNAs are DNA damage-responsive and participate in DNA damage response. For example, Schmitt AM characterized the lncRNA DINO, which regulates DNA damage signal transduction^9^. He showed that DINO is induced in doxorubicin-induced DNA damage and arrests the cell cycle by modulating p53 protein stability. Moreover, lncRNAs could mediate DNA damage repair by interacting with core protein components of DDR directly, such as PARP1 and Ku80. In response to irradiation, LncRNA LINP1 is recruited to the chromatin and enhances nonhomologous end-joining (NHEJ) activity by acting as a modular scaffold for Ku80 and DNA-PKcs^10^. These studies suggested that lncRNAs regulate signaling events involved in cell cycle and DNA damage repair, emerging as essential roles in DDR. Despite these important findings, the role of lncRNAs in DDR, particularly in radioresistance, remains elusive.

Long intergenic non-protein coding RNA, p53-induced transcript (LINC-PINT), also known as MKLN1 antisense RNA 1 (MKLN1-AS1), was initially found to serve as a tumor suppressor in colon cancer^11^, and then be bolstered strongly in pancreatic ductal adenocarcinoma, lung cancer, and osteosarcoma^12–14^. Furthermore, our previous study showed that the polymorphism of *LICN-PINT* is associated with NPC chemoradiotherapy toxicities^15^. However, LINC-PINT’s function in NPC and potential regulatory mechanisms in radiotherapy have not been revealed.

Here, we showed that LINC-PINT functioned as a tumor suppressor in NPC, and its expression was negatively related to prognosis. Moreover, LINC-PINT responded to DNA damage and reduced cell tolerance to ionizing radiation in vitro and in vivo. Mechanistically, interacting with DNA-PKcs, LINC-PINT inhibited DNA-PKcs recruitment at DNA damage sites and decreased the levels of DNA damage repair factors. Thus, we showed for the first time that LINC-PINT was involved in DDR regulation directly, and it has the potential to be a target for radiosensitization in NPC.

## Results

### LINC-PINT is downregulated and acts as a tumor suppressor in NPC

**Fig. 1:**
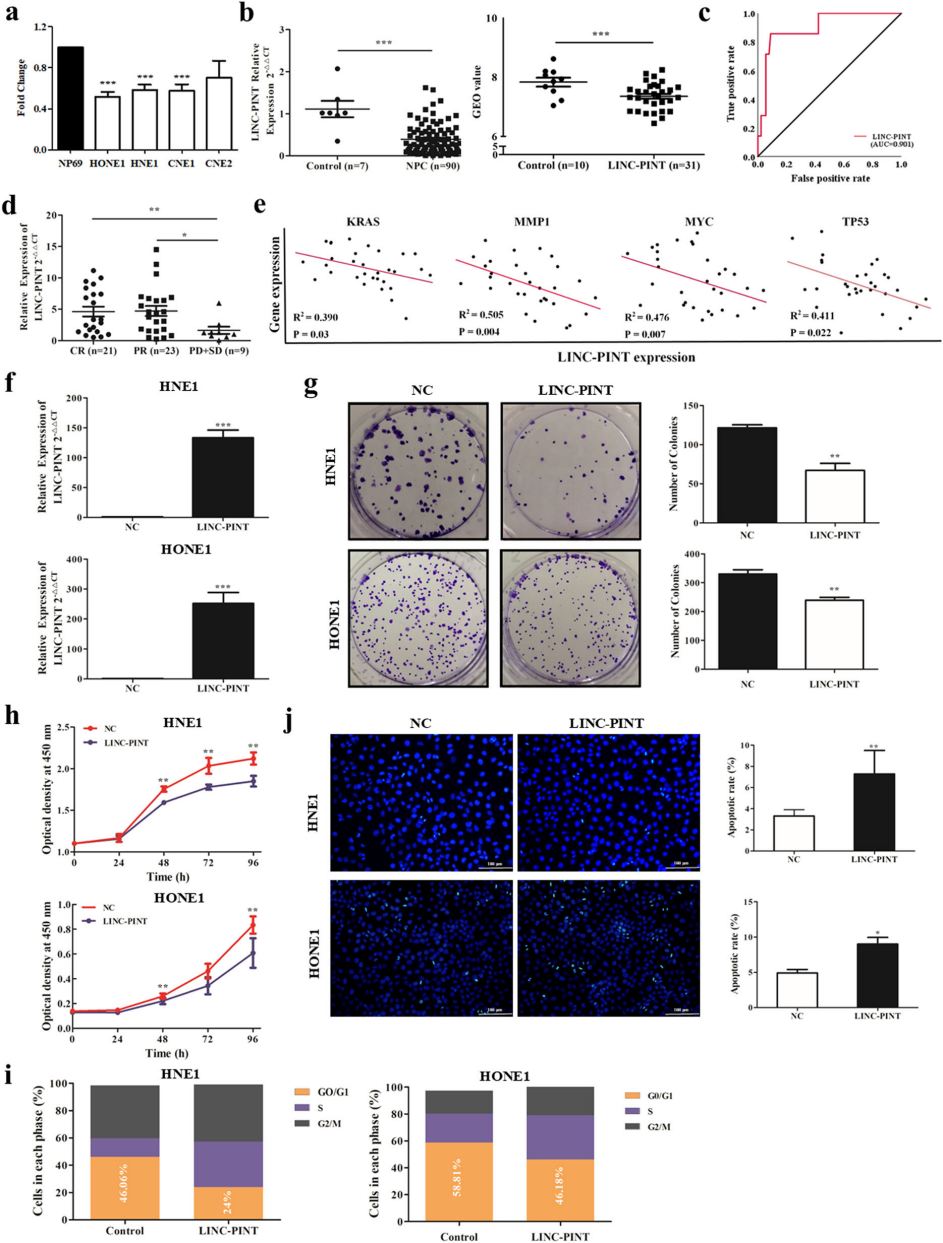
LINC-PINT is downregulated in NPC and acts as a tumor suppressor. **a** The fold change of LINC-PINT expression in NPC cells (HNE1, HONE1, CNE1, and CNE2) compared with that in immortalized nasopharyngeal epithelial cell line NP69 by qRT-PCR analysis. **b** The expression of LINC-PINT in NPC tumor tissues from our data (left) and the GDS3341 dataset (right). **c** In our data, the area under the ROC curve showing the discriminatory power of the LINC-PINT expression to predict the risk of NPC. **d** Correlation between LINC-PINT expression and treatment efficacy. CR complete response, PR partial response, SD stable disease, PD progressive disease. **e** Correlation between LINC-PINT expression and expressions of oncogenes (KRAS, MMP1, MYC, and TP53) in the NPC samples from the GDS3341 dataset. **f** HNE1 and HONE1 cells overexpressed LINC-PINT and verified by qRT-PCR. **g** LINC-PINT overexpression inhibited colony formation in NPC cells. Representative images (left) and quantitative analyses (right) are shown. **h** LINC-PINT overexpression suppressed cell proliferation in NPC cell lines, which are detected by CCK-8 assay. **i** Effect of LINC-PINT overexpression on the cell cycle progression. **j** Hoechst staining (green fluorescence) was also used to detect the effect of LINC-PINT on changes in apoptosis (up), Scale bar = 100 μm. Statistical diagrams show significant differences (bottom). Data represent the mean ± SEM from three independent experiments (**a**–**j**). Significance calculated with Student’s *T* test. *P < 0.05, **P < 0.01, ***P < 0.001. Data shown are mean ± SEM.

To understand the pathological relevance of LINC-PINT in NPC, we analyzed the GEO NPC dataset (GDS3341) and then verified the expression of LINC-PINT in NPC cell lines and clinical samples and by qRT-PCR (Fig. 1a, b). The expression of LINC-PINT was approximately 2.85-fold lower in tumor tissues (*n* = 90) than in the control group (*n* = 7), which was consistent with the analysis of NPC cell lines. Following a receiver-operating characteristic (ROC) curve-based approach, we found the expression level of LINC-PINT could distinguish cancer tissues from rhinitis tissues effectively (AUC = 0.901) based on our data (Fig. 1c). Furthermore, our results exhibited that low expression of LINC-PINT indicated poor radiotherapy efficacy in NPC patients (Fig. 1d). We further analyzed the GEO dataset (GDS3341) and found a negative correlation between LINC-PINT and some classically defined oncogenes of NPC, including KRAS, TP53, MMP1, and MYC (Fig. 1e). Taken together, these findings suggest that LINC-PINT was downregulated in NPC and may act as a tumor suppressor.

To determine whether LINC-PINT affects NPC cell growth, LINC-PINT was overexpressed efficiently in HNE1 and HONE1 cells (Fig. 1f). Ectopic expression of LINC-PINT inhibited cell proliferation and increased the S phase population in both cancer cells prominently (Fig. 1g–i). Furthermore, Hoechst staining assay results demonstrated that LINC-PINT upregulation increased the total apoptosis rate (Fig. 1j). Collectively, our data emphasized the tumor suppressor role of LINC-PINT in NPC, given decreasing cancer cell proliferation by arresting the cell cycle and increasing apoptosis.

### LINC-PINT is transcriptionally induced by DNA damage and increases radiosensitivity in vitro

**Fig. 2:**
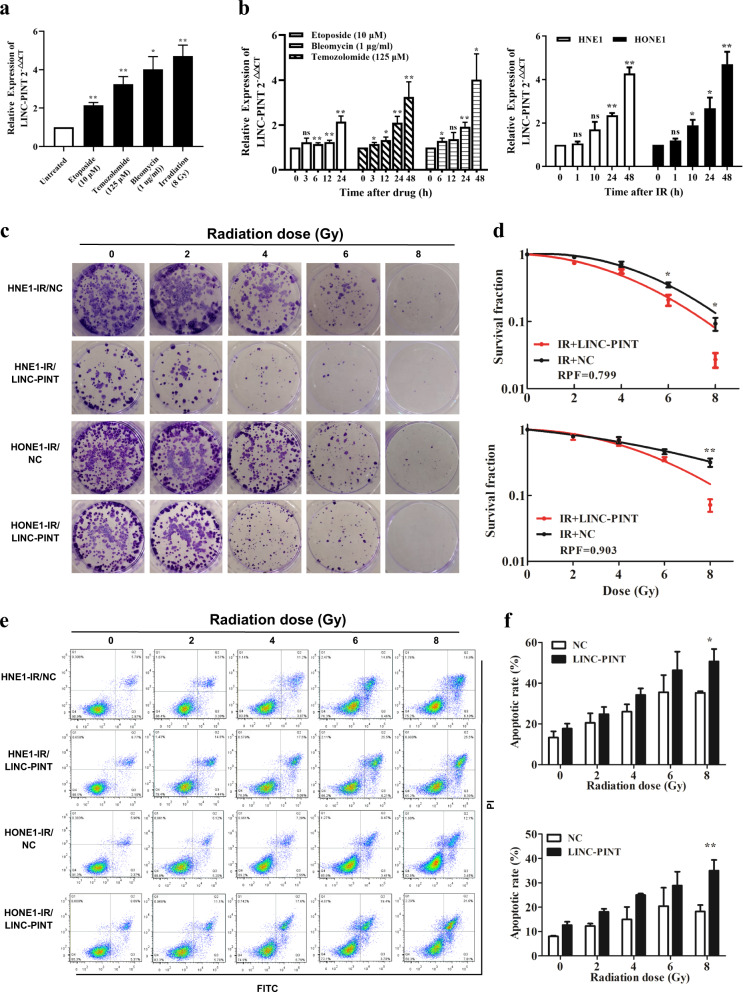
LINC-PINT is transcriptionally induced by DNA damage and increases radiosensitivity in vitro. **a** LINC-PINT was induced upon DNA damage with different DNA-damaging agents. HNE1 cells were treated with the DNA-damaging agents: Eto (10 μM), Bleo (1 μg/ml), TMZ (125 μM), and IR (8 Gy). LINC-PINT levels were measured by qRT-PCR. **b** Expression of LINC-PINT was determined by real-time RT-PCR after treating HNE1 cells with different DNA-damaging agents at different time points. RNA samples were analyzed by quantitative RT-PCR. **c** Representative images of clonogenic survival assay in HNE1 and HONE1 cells, which were transiently transfected with LINC-PINT overexpression plasmid or control plasmid, followed by a range of 0–8-Gy radiation doses. **d** It was shown that radiation survival curves were fitted by a linear quadratic equation. Up, HNE1 cells; Bottom, HONE1 cells. **e** Flow-cytometry analysis was performed to detect cell apoptosis for NPC cells, which were transfected with LINC-PINT vectors or negative controls and exposed to different doses of radiation. **f** Quantitative analyses of cell apoptosis in HNE1 (up) and HONE1 (bottom). Data represent the mean ± SEM from three independent experiments (**a**–**f**). Statistical significance was calculated using a two-sided *t* test. *P < 0.05, **P < 0.01, ***P < 0.001.

To explore whether LINC-PINT is a DNA damage-responsive lncRNA, we measured the expression level of LINC-PINT after exposing HNE1 cells with DNA-damaging agents etoposide (Eto, 10 μM), bleomycin (Bleo, 1 μg/ml), temozolomide (TMZ, 125 μM), and irradiation (IR, 8 Gy). LINC-PINT was found to be induced upon DNA damage in response to all cytotoxic agents (Fig. 2a), suggesting a global role in the DNA damage response. Besides, the induction of LINC-PINT was time-dependent. Particularly, LINC-PINT expression was increased approximately 4–5-fold in NPC cells at 48 h after irradiation (Fig. 2b).

The imbalance between proliferation and apoptosis underlies the therapeutic resistance of NPC^4^. NPC cells were exposed to different doses of radiation, and a colony-formation assay was performed. Compared with negative control, cell survival was decreased after radiotherapy in the LINC-PINT-overexpressing group (Fig. 2c). The radiation dose–response curve is a standard method to quantify the clonogenic survival assay. As shown in Fig. 2d, an enhancement of radiation sensitivity was more obvious in HNE1 cells (RPF = 0.799). Given the critical role of apoptosis in radioresistance, we analyzed the functional characteristics of LINC-PINT in irradiation-induced apoptosis. We found that high expression of LINC-PINT increased the apoptosis of radiotherapy in a dose-dependent manner (Fig. 2e, f). Compared with the control group, more than 2-fold apoptotic cells were presented in the LINC-PINT-overexpressing cells after 8 Gy irradiation (*P* < 0.05). Briefly, LINC-PINT upregulation in NPC leading to the cells more sensitive to ionizing radiation.

Next, we evaluated the effect of LINC-PINT on cell sensitivity to cisplatin, which is usually applied in concurrent radiochemotherapy for NPC patients. Unfortunately, the expression level of LINC-PINT could not be induced by cisplatin in a time-dependent manner (Supplementary Fig. S1a). The half-maximal inhibitory concentration (IC50) of cisplatin did not have obvious changes between control cells and LINC-PINT overexpressing cells (Supplementary Fig. S1b).

### LINC-PINT represses tumor growth in vivo following irradiation

**Fig. 3:**
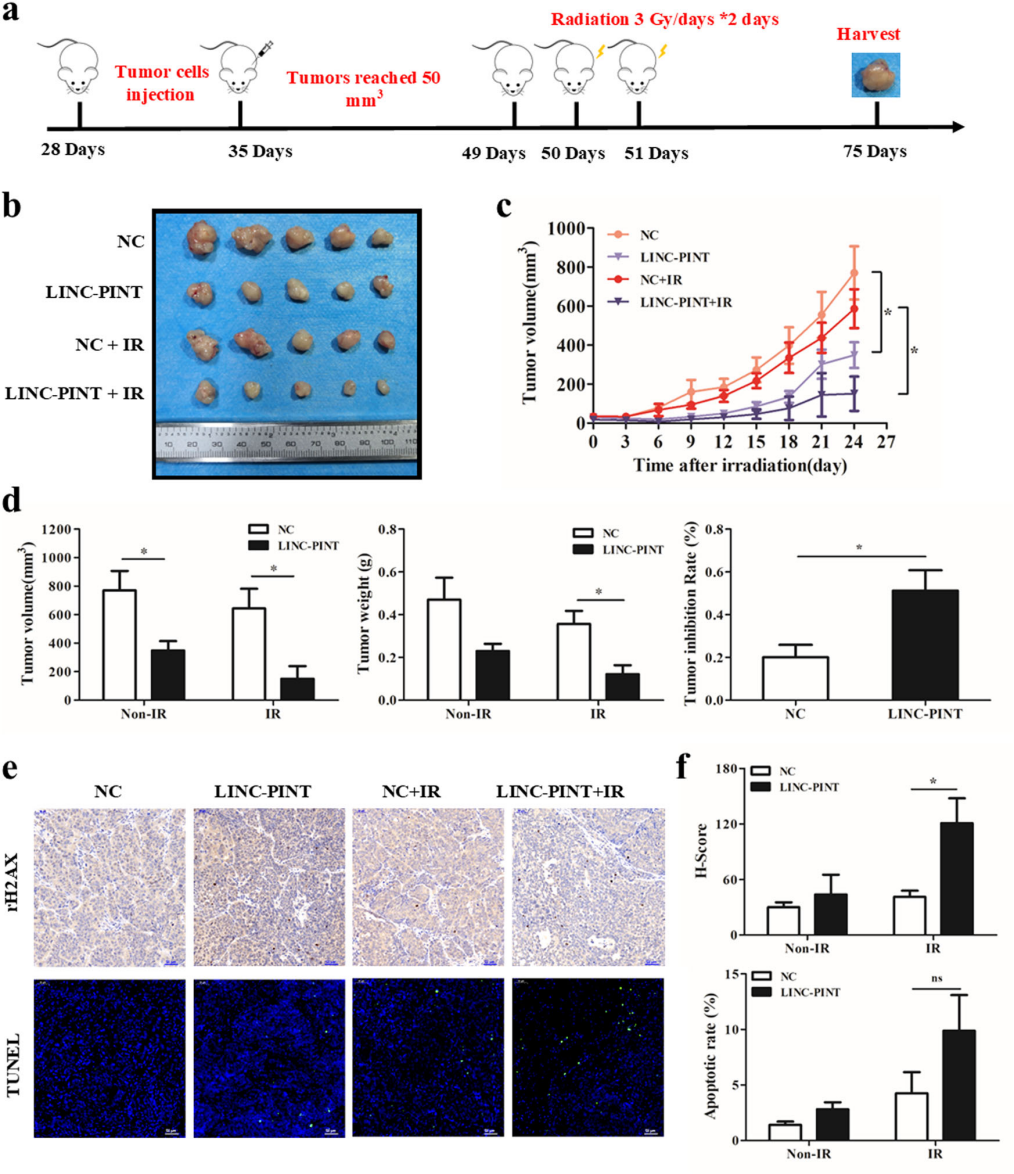
LINC-PINT is a radiotherapy sensitizer for NPC in vivo. **a** Treatment schedule in vivo. **b** Tumor images of xenografts in four groups at the end of the experiment. **c** Tumor growth curves of xenografts following initiated treatment with irradiation. Each tumor size was monitored and measured three times per week. **d** The volume (left), weight (middle), and the tumor inhibition rate (right) of xenograft tumors at sacrifice were analyzed. **e** Representative images of immunohistochemical staining for γH2AX (up) and TUNEL staining for apoptosis (bottom) in LINC-PINT-overexpressing tumors and control group with or without irradiation. Scale bar = 50 μm. **f** Quantification of γ-H2AX staining (up) and TUNEL staining (bottom) in four groups. Data represent the mean ± SEM. Statistical significance was calculated using a two-sided *t* test. *P < 0.05, **P < 0.01, ***P < 0.001.

The treatment schedule of xenograft tumor models was shown in Fig. 3a. The tumors grew slower and showed a more pronounced antiproliferation effect in LINC-PINT overexpressed group post irradiation (Fig. 3b, c). The tumor inhibition rate in the LINC-PINT-overexpressing group was 51.2%, which was significantly higher than 20.1% in the control group (Fig. 3d). Consistently, tumors with high expression of LINC-PINT appeared to have more apoptotic cells, especially in the combination group (Fig. 3e, f). By immunohistochemical staining in tumors, we found that LINC-PINT upregulation caused increased positive cells of γH_2_AX noticeably with or without ionizing radiation (Fig. 3e, f). Together, our observation indicated that LINC-PINT would sensitize tumors to radiotherapy in vivo, which attributed to DNA damage and apoptosis partially.

### LINC-PINT upregulation impairs DNA damage repair

**Fig. 4:**
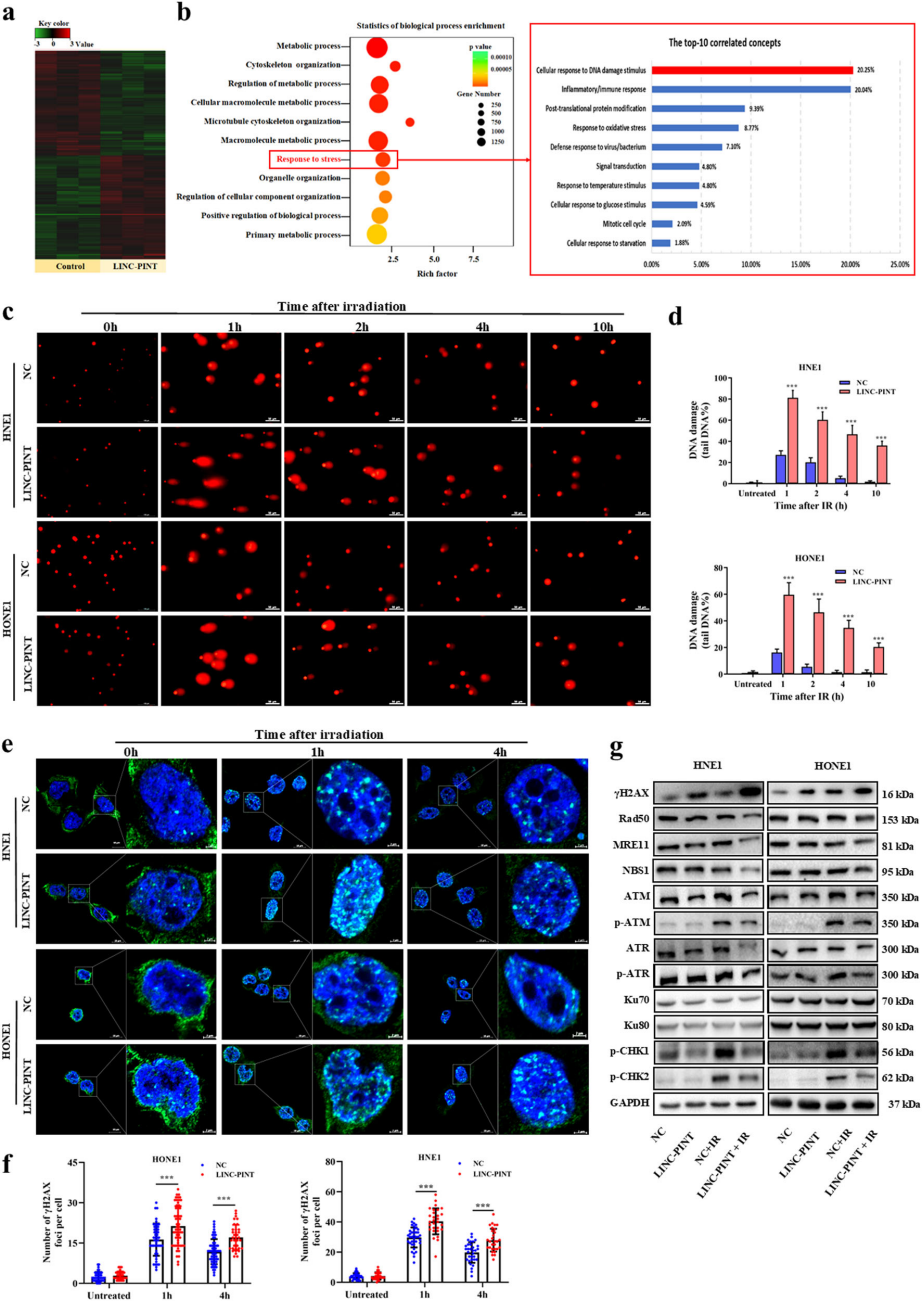
LINC-PINT participates in DNA damage repair response. **a** Heatmap of transcriptomic profiling in HNE1 cells transfected with LINC-PINT-expressing plasmid or control plasmid (green = downregulated; red = upregulated). **b** Gene ontology analysis of LINC-PINT-affected mRNA. The “Response to stress” network was among the top ten most differentially expressed. **c**, **d** IR-induced DNA damage in LINC-PINT-overexpressing cells and control group were analyzed by the comet assay at oh, 1, 2, 4, and 10 h. Representative images (**c** scale bars, 50 µm) and quantitative analysis (**d**) of tail DNA in each group. **e**, **f** Representative fluorescence images (**e** scale bars, 20 µm; green, γ-H2AX; blue, DAPI) and quantification (**f**) of γ-H2AX foci in cells with and without LINC-PINT overexpression. H2AX foci were counted at 0, 1, and 4 h post irradiation. **g** The expression of different DNA damage response proteins in HNE1 and HONE1 cells, which were transfected with LINC-PINT-overexpressing plasmid or control plasmid with and without irradiation. The protein level was analyzed by western blot more than three times. GAPDH was used as a loading control. Data represent the mean ± SEM from three independent experiments (**c**–**g**). Statistical significance was calculated using a two-sided *t* test. *P < 0.05, **P < 0.01, ***P < 0.001.

LncRNA transcripts often regulate gene expression directly or indirectly^16^. To explore the underlying mechanism by which LINC-PINT increased radiosensitivity in NPC cells, we performed transcriptome microarray profiling in HNE1 transfected with negative control or LINC-PINT expression vector. Using microarray analysis, we found several upregulated or downregulated genes (Fig. 4a). GO pathway analysis revealed that the stress response pathway (GO:0006950) among the top 10 processes affected by LINC-PINT (*P* = 5.11e^−06^) (Fig. 4b), indicating that it may play a key role in the regulation of DNA damage stimulus in NPC.

To further test our hypothesis, we investigated whether LINC-PINT-mediated radiosensitization may through decreasing DNA damage repair progress. In a DNA comet assay, the comet tail was detected at various time points after treatment with 8 Gy irradiation. As shown in Fig. 4c, ionizing radiation-induced comet formation in both NPC cells, this damage was repaired rapidly in a time-dependent manner. However, the LINC-PINT-overexpressing cells displayed significantly increased tail length across all time points, with the most pronounced effect at 1 h. Compared with control cells, there is still 20–36% damaged DNA in LINC-PINT-overexpressing cells after 10 h of recovery following irradiation (Fig. 4d), suggesting that LINC-PINT either enhanced DNA damage burden or decreased DNA-repair efficiency.

Next, we used an immunofluorescence assay to monitor the formation of γH2AX foci, which is a quantitative indicator for unresolved dsDNA damage^17^. Consistent with the comet assays, LINC-PINT-overexpressing cells exhibited noticeably higher levels of γH2AX positive foci during a 4 h period following irradiation, particularly at 1 h after exposure to irradiation (Fig. 4e, f). Over time, cells repaired the IR-induced DSBs more efficiently upon ectopic LINC-PINT expression. And the protein level of γH2AX was further confirmed by western blot both with and without irradiation treatment (Fig. 4g). These demonstrate that LINC-PINT upregulation could induce more DNA damage.

ATR and ATM can phosphorylate H2AX at serine 139 in response to DNA damage, and then promotes the formation of γH2AX foci^18^. Thus, we want to investigate whether LINC-PINT can inhibit ATM/ATR signaling and reduce H2AX phosphorylation. As shown in Fig. 4g, western blotting assays demonstrated that LINC-PINT significantly inhibited phosphorylation of ATM/ATR upon DNA damage with altering total ATM/ATR. CHK1 and CHK2, typical substrates of the ATM and ATR kinases, collaborate to regulate cell cycle arrest in cells with DNA damage. In our results, p-CHK1 and p-CHK2 were induced after irradiation treatment, which was largely abolished in LINC-PINT-overexpressing cells. In addition, we explored the underlying mechanisms by which LINC-PINT inhibited ATM/ATR activity. As expected, higher levels of LINC-PINT in HNE1 and HONE1 cells with lower expression of major DNA-repair pathway executors, such as RAD51, NBS1, and MRE11. However, Ku70, Ku80, and apoptosis proteins such as Bcl2, Bax, and PARP were not affected by LINC-PINT (Fig. 4g and Supplementary Fig. S1c). Collectively, the above evidence supported that LINC-PINT enhanced radiosensitivity by increasing DNA damage as well as impairing DNA damage repair.

### LINC-PINT targets DNA-PKcs directly

**Fig. 5:**
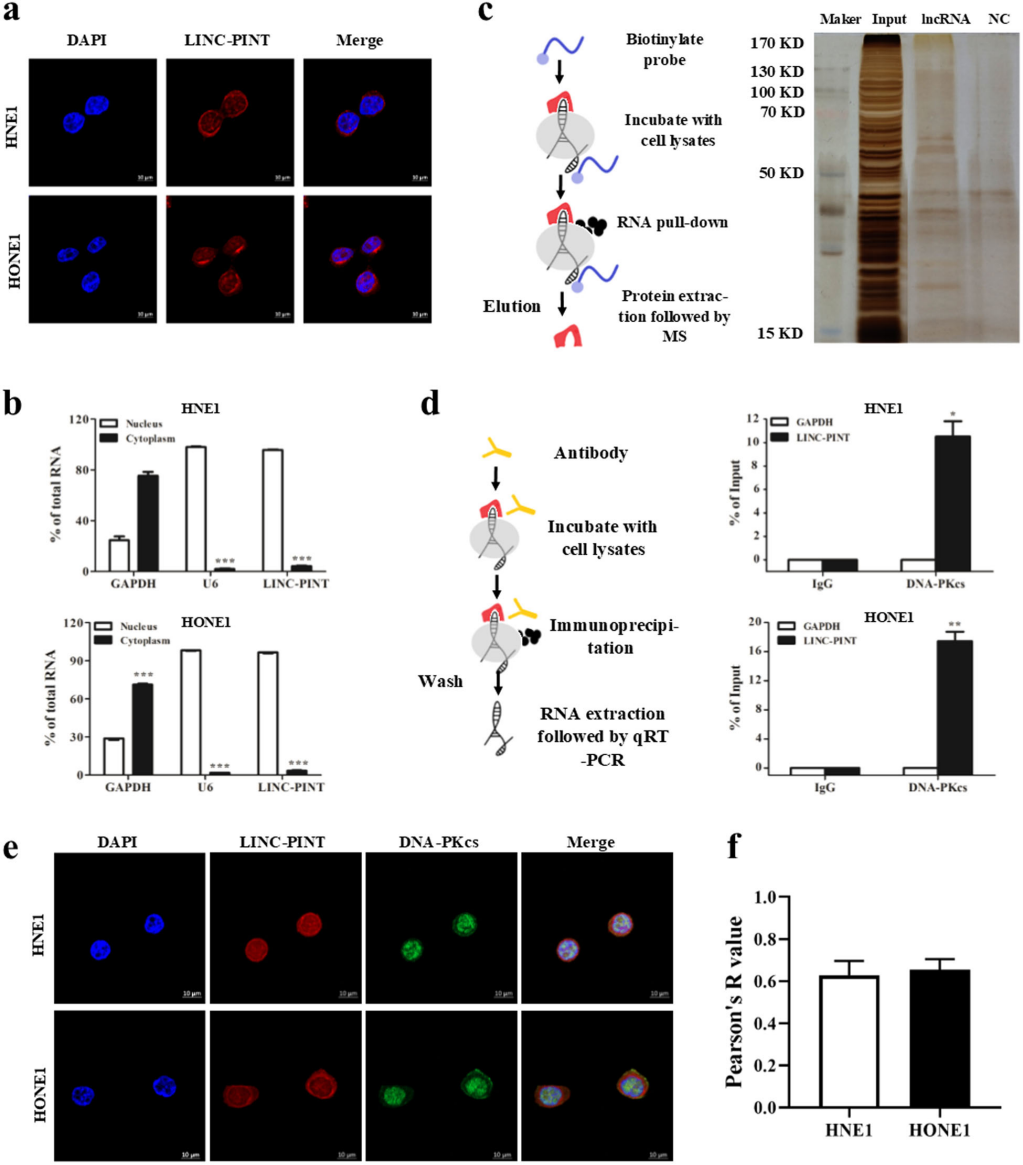
DNA-PKcs is a direct target of LINC-PINT. **a** Representative images of the FISH assay for detecting the subcellular localization of LINC-PINT in HNE1 and HONE1 cell lines (scale bar, 10 μm; blue, DAPI; red, LINC-PINT). **b** The expression of LINC-PINT in cytoplasmic and nuclear fractions of NPC cells. RNA samples were analyzed by qRT-PCR three times. Data represent the mean ± SEM. Statistical significance was calculated using a two-sided *t* test. *P < 0.05, **P < 0.01, ***P < 0.001. **c** Identification of the binding proteins of LINC-PINT in HNE1 and HONE1 cells. Left, Schematic diagram of the RNA pull-down strategy. Right, results of Silver staining. **d** RIP was performed using anti-DNA-PKcs antibodies. Left, schematic diagram of the RIP assay. Right, the levels of LINC-PINT and GAPDH in the coprecipitates were determined by qRT-PCR. **e**, **f** Representative fluorescence images (**e** scale bars, 10 µm; blue, DAPI; r**e**d, LINC-PINT; green, DNA-PKcs) and quantification (**f** *n* = 3 biologically independent samples) of colocalization between LINC-PINT and DNA-PKcs in HNE1 and HONE1 cells. Pearson’s R values were analyzed by Image J.

The specialized functions of lncRNA depend on its subcellular localization^19^. As presented in Fig. 5a, LINC-PINT mainly locates in the nucleus. To further validate the subcellular localization of LINC-PINT, cytoplasmic and nuclear RNA fractionation assay was employed. In contrast to GAPDH but similar to U6, LINC-PINT is mainly located in the cell nucleus (Fig. 5b). Nuclear-localized lncRNAs may function as modular to interact with specific proteins^19^. We used RNA pull-down assay followed by mass spectrometry to identify the targets of LINC-PINT. Fortunately, DNA-PKcs, a decisive factor involved in NHEJ, was a candidate LINC-PINT-associated protein in NPC cells (Fig. 5c and Supplementary Fig. S1d). We further validated the result through the RNA-IP experiment and found LINC-PINT was abundant in DNA-PKcs IP samples (*P* < 0.05) (Fig. 5d and Supplementary Fig. S1e). In line with these data, immunofluorescence staining assay found that DNA-PKcs signals were colocalized with LINC-PINT signals (Fig. 5e, f). Consequently, we inferred that LINC-PINT is a special factor to interact with DNA-PKcs.

### LINC-PINT regulates DNA-PKcs post-transcriptionally

**Fig. 6:**
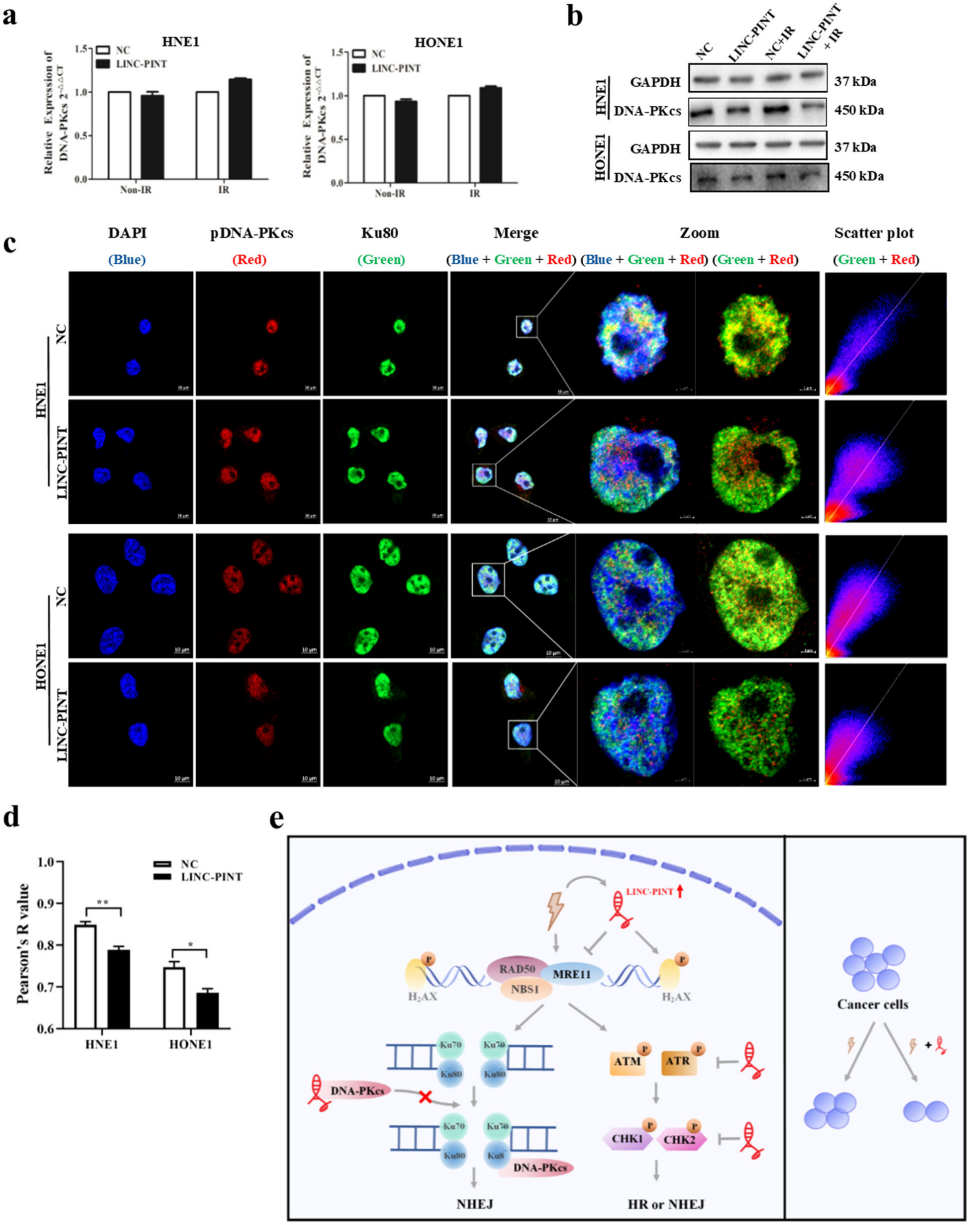
Mechanism of the radiosensitizing effect of LINC-PINT. **a**, **b** LINC-PINT modulated the protein level of DNA-PKcs. After transfection, cells were treated with or without 8-Gy radiation and subjected to qRT-PCR (**a**) and western blot (**b**) for DNA-PKcs. Data represent the mean ± SEM from three independent experiments. Statistical significance was calculated using a two-sided *t* test. *P < 0.05, **P < 0.01, ***P < 0.001. **c**, **d** Representative fluorescence images (**c** scale bars, 10 µm; blue, DAPI; red, pDNA-PKcs; green, Ku80) and quantification (**d** *n* = 3 biologically independent samples) of colocalization between pDNA-PKcs (Ser2056) and Ku80 in HNE1 and HONE1 cells. Pearson’s R values were analyzed by Image J. **e** The molecular pathway of LINC-PINT-mediated radiosensitization. On the one hand, LINC-PINT was responsive to DNA damage, inhibiting DNA damage repair through ATM/ATR-Chk1/Chk2 signaling pathways. On the other hand, LINC-PINT increased radiosensitivity by interacting with DNA-PKcs and negatively regulated the expression and recruitment of DNA-PKcs. Therefore, LINC-PINT may confer radiosensitivity to NPC.

To recognize the underlying mechanism of LINC-PINT working on DNA-PKcs, we detected the expression of DNA-PKcs both in the level of mRNA and protein. As shown in Fig. 6a, b, LINC-PINT downregulated the expression of DNA-PKcs at the protein level, rather than the mRNA level. Phosphorylation of the Ser-2056 residue of DNA-PKcs would affect the affinity and activity of DNA-PKcs in response to DNA damage^20^. Such phosphorylation-induced alteration is a Ku80-dependent process^20^. Therefore, we used immunofluorescence assay to investigate if LINC-PINT did or not regulate DNA-PKcs recruitment in response to DNA damage. Our results showed that LINC-PINT overexpression resulted in a decreased colocalization coefficient between pDNA-PKcs (Ser2056) and Ku80 (Fig. 6c, d), suggesting that LINC-PINT had a high degree of selectivity in inhibiting DNA repair by negatively regulated the recruitment of pDNA-PKcs.

## Discussion

Radiotherapy is a central pillar of NPC standard therapy that delivers anti-tumor effects through causing DNA lesions. Therefore, cellular DNA damage response is a key factor in determining patients’ outcomes following radiotherapy^5^. However, most studies about DDR have focused on proteins^21,22^, the roles and functions of lncRNAs in DNA-repair machinery remain poorly understood. Here, using cell culture and mouse xenograft models, we identified the radiosensitizing effect of LINC-PINT and characterized its role in the DNA damage response pathway. As a radiosensitizing lncRNA, LINC-PINT has three characteristics: (i) LINC-PINT acted as a tumor suppressor to mediate cell cycle arrest and apoptosis; (ii) LINC-PINT was responsive to DNA damage, inhibiting DNA damage repair through ATM/ATR-Chk1/Chk2 signaling pathways; (iii) LINC-PINT interacted with DNA-PKcs, which is a key molecule in initiating DDR cascades (Fig. 6e).

LINC-PINT functions as a tumor suppressor and is downregulated in multiple cancers. For example, LINC-PINT inhibits cell proliferation and promotes apoptosis in colon cancer^11^. Lower levels of the LINC-PINT are significantly associated with a poorer prognosis in lung cancer^13^. Consistent with what other investigators have reported, our data demonstrated that LINC-PINT impaired the ability of NPC cells to proliferate and survive. Interestingly, we revealed a new function of LINC-PINT in NPC radiosensitivity. Following DNA damage, cells activate DDR to arrest the cell cycle for essential DNA repair and can lead to lethality if unrepaired^4,23^. Therefore, the cell cycle is a key factor that influences radiosensitivity. Here, we showed that LINC-PINT overexpression increased the percentage of cells in the S and G2 phases significantly. This suggests that LINC-PINT as a regulator of the cell cycle may also likely to be an important player in DDR, as most cells are radiosensitive in the late G2 (refs. ^24–26^). It is reasonable to propose that reactivates the tumor-suppressive role of LINC-PINT in cancer cells may arrest the cell cycle and then reach the therapeutic threshold^27^.

Ionizing irradiation is used in medicine as a cancer treatment because it causes DNA damage to arrest the cell cycle and induce apoptosis. Any increased DNA-repair capacity can lead to irradiation resistance and limits the efficacy of radiotherapy. Therefore, DNA damage repair inhibitors have been investigated as clinical agents to increase radiosensitivity, and consequently increase the efficacy of radiotherapy^4,23^. LINC-PINT expression level was upregulated in response to different DNA-damaging agents, including ionizing radiation, playing a global role in DDR. In response to irradiation, LINC-PINT was induced, and the elevated LINC-PINT expression caused significant and persistent DNA damage. Ectopic LINC-PINT expression in NPC cells led to a significant increase in the length of the comet tail and nuclear γH2AX foci across all time points, representing an increase in DNA breaks degree. In summary, this DNA damage repair progress was significantly delayed. LINC-PINT has an impact on DNA-repair kinetics, making NPC cells more prone to radiation-induced DNA damage and therefore more vulnerable to radiation. Further work could investigate the more precise mechanism of LINC-PINT in DNA damage repair progress.

Recognizing the DNA lesions is an early event in DSB signaling before repair. DNA-PK complex, a sensor to detect DNA damage, includes three components, namely Ku70, Ku80, and DNA-PKcs^28,29^. Distinct from the MRN complex that regulates both homologous recombination (HR) and NHEJ pathways, DNA-PK is necessary for NHEJ. Once DSBs occur, Ku70/Ku80 complex encloses the DNA ends and recruits catalytic subunit DNA-PKcs is the initial step in NHEJ^28,29^. Here, we demonstrated that LINC-PINT impaired DNA damage repair, in part, by targeting DNA-PKcs directly and inhibiting its expression at the protein level. DNA-PKcs is a key molecule in initiating DDR cascades. If DNA-PKcs get mutation or deficiency, it would fail to restore DSB caused by ionizing radiation and increase the radiosensitivity finally^30^. Changes with the phosphorylation status of DNA-PKcs would influence DNA-end ligation and the recruitment of downstream repair factors^20,31,32^. Ser2056 is generally used to examine autophosphorylation, as the marker for DNA-PKcs activity^20^. Ku80, a specific protein co-factor for DNA-PKcs, would enhance the binding affinity of the catalytic subunit^20,32–34^. In our study, although LINC-PINT did not affect the expression of Ku80, we also provided evidence that LINC-PINT decreased the colocalization between pDNA-PKcs and Ku80. We hypothesized that LINC-PINT would serve as a decoy for DNA-PKcs to increased radiation sensitivity. However, further studies are necessary to validate this hypothesis.

ATM and ATR are the other two master regulators of the DDR. At DNA lesions, the MRN complex activates and recruited ATM/ATR which then activates a DDR^30,31^. Following radiation, ATM phosphorylates several substrates to orchestrate DNA repair, checkpoint signaling, cell survival, and death^35^. CHK2 is a well-characterized substrate of ATM. The expressions of ATM and CHK2 have been connected to clinical prognosis. It has been demonstrated that ataxia-telangiectasia patients with ATM mutation are sensitive to irradiation^36^, suggesting that inhibition of ATM can sensitize cancer cells to radiotherapy. Reduced expression levels of ATM-CHK2 were associated with radio- or chemo-sensitivity and in lung cancer^37^, colon cancers^38^, and gliomas^39^. Although ATM seems to be an apical kinase in initiating DDR cascades, ATR is also necessary for DNA damage repair by phosphorylating and activating the protein kinase CHK1. ATR and CHK1 phosphorylate several proteins involved in HR signaling^4,25^. Similarly, high ATR-CHK1 expressions are markedly correlated to poor outcomes of radiotherapy in lung cancer^37,40^ and breast cancer^41^. Consistently, we found that increased expression of LINC-PINT inhibited the MRN complex and ATM/ATR-CHK1/CHK2 axis significantly, which may underline mechanisms of how LINC-PINT sensitized NPC cells to irradiation in vitro and in vivo.

To summarize, our study has revealed a new function of LINC-PINT in NPC radiosensitivity. Overexpression of the LINC-PINT impairs DNA damage repair, leading to DNA damage accumulated and rendering the tumor cells susceptible to ionizing radiation. Mechanistically, LINC-PINT was responsive to DNA damage, inhibiting DNA damage repair through ATM/ATR-Chk1/Chk2 signaling pathways. Moreover, LINC-PINT increased radiosensitivity by interacting with DNA-PKcs and negatively regulated the expression and recruitment of DNA-PKcs. Therefore, small molecules that restore LINC-PINT expression or facilitate LINC-PINT–DNA-PKcs interactions could be useful to reverse radioresistance in NPC.

## Materials and methods

### Tumor specimens

Ninety fresh tissue samples were collected from the individuals, who were diagnosed with NPC histopathologically and then enrolled in Hunan Provincial Cancer Hospital. We also acquired seven cases of rhinitis tissue biopsies in the same period. All tissue samples were obtained at the time of diagnosis before radiotherapy and chemotherapy, then stored at −80 °C for further analysis. This study was performed after approval by the Independent Ethical Committee of Institute of Clinical Pharmacology, Central South University (CTXY-140007-2) and was conducted following the Declaration of Helsinki. And we obtained informed written consent from all subjects. The efficacy of radiotherapy was evaluated clinically for primary lesions according to magnetic resonance imaging (MRI) three months after radiotherapy. The clinicopathologic parameters of the NPC patients are shown in Supplementary Table S1.

### Cell culture

Four human nasopharyngeal carcinoma cell lines, HONE1, HNE1, CNE1, CNE2, and a normal nasopharyngeal epithelial cell line, NP69, were obtained from the Advanced Research Center of Central South University with further authentication. Cancer cell lines were maintained in RPMI 1640 medium (Invitrogen, Carlsbad, CA), which were supplemented with 10% fetal bovine serum (Invitrogen, Carlsbad, CA), penicillin (100 units/ml), and streptomycin (100 units/ml). According to a standard protocol, NP69 was cultured in a keratinocyte serum-free medium (Invitrogen, Carlsbad, CA) containing 10% fetal bovine serum. All cells grew in an incubator with 5% CO_2_ under a constant temperature.

### Plasmid construction and transfection

For overexpression, Complementary DNA (cDNA) of lncRNA LINC-PINT was synthesized and cloned into the multiple cloning sites in the GV219 expression vector (Genechem, Shanghai, CHN). The empty vector was used as a negative control. Plasmids were expanded in competent *Escherichia coli* (Stbl3) and extracted using the Endofree Plasmid Maxi Kit (Cat no: 12362, Qiagen, GER) according to the manufacturer’s protocol. And then sequencing was performed to verify the constructs. When cell confluency reaches 70–80%, the cells were transfected with 1 μg of plasmid DNA using Lipofectamine 3000 (Invitrogen, USA). The cells were collected for further researches 24 h after transfection. LINC-PINT overexpression efficacy was confirmed by qRT-PCR.

For lentiviral construction, plasmids were also developed by Shanghai Genechem Co., Ltd. Shanghai, China. LINC-PINT cDNA was inserted into the GV348 lentivirus vector and packaged into lentivirus. NPC cells were infected with virus plasmids and screened with puromycin at a concentration of 1 μg/mL. qRT-PCR was performed to detect LINC-PINT expression.

### RNA isolation and quantitative real-time-PCR (qRT-PCR)

According to the manufacturer’s protocol, RNA was isolated from nasopharyngeal cancer cells or tissues using RNAiso reagent (Takara Bio Inc., Japan). The quality and amount of RNA were determined with an ultraviolet spectrophotometer (Thermo Scientific, Rockford, IL). Then a total of 1000 ng RNA was prepared to reverse transcription using a PrimeScript^TM^ RT reagent Kit (Takara Bio Inc., Japan). qPCR was carried out in triplicates by Roche LightCycler 480 system (Roche, Basel, Switzerland). The LINC-PINT expression level was calculated by 2^−ΔΔCt^ method against GAPDH or U6 for normalization. Amplification procedure was listed as follows: denaturation at 95 °C 30 s; PCR at 95 °C 5 s and 60 °C 30 s, for 40 cycles; anneal at 95 °C 5 s and 65 °C 1 min. The primer sequences were listed as follows: (1) GAPDH-F: 5’-ACAACTTTGGTATCGTGGAAGG-3’ and GAPDH-R: 5’-GCCATCACGCCACAGTTTC-3’; (2) U6-F: 5’-GCTTGCTTCAGCAGCACATA-3’ and U6-R: 5’-AAAAACATGGAACTCTTCACG-3’; (3) LINC-PINT-F: 5’-TCATCTATCAGCCCATTACAC-3’ and LINC-PINT-R: 5’-AAGTTATGCCACAAATACCAG-3’.

### Antibodies and western blotting

We performed western blotting according to a standard protocol. Proteins were collected and lysed from NPC cells by RIPA buffer (Beyotime, CHN), which contained protease inhibitor (Beyotime, CHN) and phosphatase inhibitor (Beyotime, CHN). Protein concentrations were measured by BCA kit (Beyotime, CHN). Subsequently, 10 μg samples were run on 4–12% polyacrylamide gels and then electrophoretically transferred to PVDF membranes (Millipore, USA). The membranes were blocked with 5% nonfat dry milk for 2 h at room temperature. Furthermore, samples were incubated overnight at 4 °C with primary antibodies. The next day, the membranes were incubated with secondary antibodies such as goat anti-rabbit antibody or goat anti-mouse antibody (Sigma, USA) for 2 h. Finally, protein signals were visualized by an enhanced chemiluminescence detection reagent (Invitrogen, USA) and analyzed by the Image lab software. GAPDH expression was used for normalization, and three independent experiments were done. The information about antibodies is shown in Supplementary Table S2.

### Cell proliferation assays

For CCK-8 assays, HNE1 and HONE1 cell lines were transfected with the LINC-PINT-expressing vector or control vector for 24 h. Following the manufacturer’s instructions, a density of 5.0 × 10^3^ cells/well were transferred to a 96-well plate, and cell proliferation curves were constructed every 24 h by Cell Counting Kit-8 (Selleck, USA). The absorbance at 450 nm was measured by Microplate Reader (Bio Tek, USA).

For colony-formation assays, NPC cells were transfected with the LINC-PINT-expressing vector or control vector, and then a density of 1.0 × 10^3^ NPC cells was seeded in six-well plates. When macroscopic clones appeared after incubation for 14–21 days, we removed the medium, fixed, and stained cells with 4% paraformaldehyde (Beyotime, CHN) and 0.5% crystal violet (Solarbio, CHN), respectively. Assays were done in triplicate.

For clonogenic survival assay, both HNE1 and HONE1 cells were plated in six-well plates at a density of 2.0 × 10^3^ cells/plate, which were treated with or without a range of radiation doses (0, 2, 4, 6, or 8 Gy) by the X-ray irradiator. After cultured 10–14 days, colonies comprising >50 cells were stained and counted successively. Radiation dose–response curves were constructed by GraphPad Prism 5.0. The Area Under Curve (AUC) is defined as an inactivation dose (MID). Radiation protection factor (RPF) = the MID of the test cells/the MID of control cells. Three independent experiments were performed.

### Flow-cytometry analysis of cell apoptosis and cell cycle

NPC cells were transfected with the indicated plasmids for 24 h and then treated with or without irradiation. For cell apoptosis, we collected the supernatant and trypsinized the adherent cells 24 h after various radiation doses. The collected cells were washed with phosphate-buffered saline (PBS) and then centrifuged at 3000 rpm for 10 min. Next, we stained cells by fluorescein isothiocyanate-labeled annexin V and propidium iodide according to the protocol of Annexin V-FITC Apoptosis Detection Kit (Cat no.: C1063, Beyotime, CHN). Apoptotic cells were measured by a flow cytometer (Backman, USA) and analyzed by Flowjo software. Three separate experiments were carried out.

For the cell cycle, we harvested cells 24 h after transfection and then fixed cells with 70% cold ethanol for 12–24 h at 4 °C. Cells were suspended in 1 mL PBS and then resuspended in PI/RNase Staining Solution buffer (Cat no.: C1052, Beyotime, CHN) for 15 min at room temperature. Cell cycle was subsequently detected by flow cytometry (Backman, USA), using 488-nm excitation. Three separate experiments were carried out.

### Hoechst 33258 staining

NPC cells were seeded in a six-well plate and transfected with plasmids as indicated for 48 h. The transfected cells were fixed with 4% paraformaldehyde for 10 min at room temperature. Furthermore, the cells were loaded with fluorescent dye Hoechst 33258 solution (Cat no.: C1017, Beyotime, CHN) for 5 min in the dark. After that, cells were washed with PBS three times. Morphological features of the nucleus were observed by an inversion fluorescence microscope (Nikon, Japan). Three separate experiments were carried out. We randomly captured at least three regions of the picture and calculated the number of apoptotic cells.

### RNA FISH

RNA fluorescence in situ hybridization (RNA-FISH) assay was carried out to confirm the subcellular localization and expression of lncRNA LINC-PINT in NPC cells. Briefly, the cells were inoculated in the confocal dish (Cat no.: 801002, NEST, CHN) and cultured for 24 h. After washing by PBS, the NPC cells were fixed in a 4% polyformaldehyde and then permeabilized in 0.5% Triton X-100 for 5 min. Next, cells were incubated with a pre-hybridization buffer at room temperature for 30 min in the dark. After that, a hybridization step was performed with 100 μL hybridization solution containing 2.5 μL 20 μM lncRNA FISH Probe Mix at 42 °C overnight. Following the hybridization, cells were washed with saline sodium citrate (SSC) containing 0.1% Tween-20 at 42 °C (4× SSC three times, 2× SSC three times, and 1× SSC one time) followed by dyed the nucleus with DAPI. Finally, an anti-fluorescence quenching agent was added to sealed cells. The images were captured using a fluorescence microscope equipped with a 555-nm laser.

### Cytoplasmic and nuclear RNA fractionation

Cytoplasmic and nuclear fractions were separated using PARIS^TM^ Kit (Cat no.: AM1921, Life, USA) according to the protocol. Briefly, about 1 × 10^7^ NPC cells were trypsinized and washed with ice-cold PBS. Next, 500 μL precooled cell fractionation buffer was added, and then cells were incubated on ice for 5 min. By centrifuging at 500×*g* for 5 min, we obtained the precipitation (nuclear fraction) and the supernatant (cytoplasmic fraction). The supernatant fraction was transferred into a new collection tube for further treatment, and the nuclear fraction was treated with 300 μL additional fractionation buffer to remove residual contamination. For obtaining nuclear and cytoplasmic fractions, 500 μL of lysis buffer was added and the tube was inverted five times. After that, the nucleus and cytoplasm lysate were separately mixed with an equal volume of 100% ethanol and pelleted with 14,000 rpm for one minute. After centrifuging and discarding the flow-through, the samples were rinsed in a 700-μL wash solution. The pure nucleus and cytoplasm RNA were eluted with a 40–60 μL elution solution at 100 °C. The expression of LINC-PINT was determined by RT-qPCR, and GAPDH and U6 were used as markers of cytoplasm and nucleus, respectively.

### Comet assays

Ionizing radiation-induced DNA damage was determined by comet assay. After transfection, NPC cells were radiated (8 Gy) and then harvested at 0, 1, 2, 4, and 10 h. In total, 1 × 10^3^ cells were resuspended with 10 μL PBS and then mixed with 0.8% low-melting agarose on comet slides. To allow DNA to unwind, the slides were treated with lysis solution and soaking with electrophoresis buffer for 20 min. After conducting electrophoresis at 25 V for 20 min, we neutralized the slides with Tris-HCl (pH = 7.5) and added 50 μL ethidium bromide (30 µg/mL) per slide. Finally, one hundred comets were scored per sample by using an Olympus IX71 fluorescent microscope. Three separate experiments were carried out, and statistical significance was analyzed by Student’s *t* tests.

### Tumor xenografts

Four-week-old female BALB/c nude mice were purchased and maintained under standard conditions in the Experimental Animal Center of Central South University (Hunan, China). HNE1 cells were transfected with lentivirus vectors containing GV348-LINC-PINT or GV348-Control and then selected in puromycin. A total of 1 × 10^7^ cells was suspended in 200 μL PBS and subcutaneously injected in the left axilla of the mice. Mice were randomly assigned to irradiation or non-irradiation groups (*n* = 5 mice per group) when tumors grow to ~50 mm^3^. In the radiation treatment group, tumors were exposed to fractionated irradiation, 3 Gy per fraction for 2 days. And the other group was defined as a control. Tumors’ size was monitored and measured three times per week. The volumes were calculated according to the formula: volume = length × width^2^/2. Every mouse was sacrificed 4 weeks after radiation treatment. The tumors were harvested, photographed, weighed, and embedded in paraffin for further analysis. Our animal experiments were approved by the Animal Ethics Committee of the Third Xiangya Hospital of Central South University and carried out with the US National Institute of Health Guidelines for Use of Experimental Animals.

### Immunohistochemical staining

The distribution and expression of γH2AX in paraffin-embedded tumor tissues of xenograft mice were detected by immunohistochemistry. Tissues were deparaffinized and rehydrated, then the antigen was retrieved according to standard instructions. Next, the slides were blocked with 3% blocking buffer and incubated with the anti-γH2AX antibody at 4 °C overnight, followed by secondary antibody and counterstained with 10% hematoxylin. Finally, images were captured at ×20 magnification using a Pannoramic MIDI (3D HISTECH, Budapest, Hungary). Staining signals were scored base on a formula as follows: H-score = ∑ (Pi * i), Pi = the proportion of positive cells, i = staining intensity. Three images were evaluated.

### TUNEL staining

TUNEL Apoptosis Assay Kit (Beyotime, China) was used to detect apoptotic cells of paraffin-embedded tissue. According to the protocol, tissues were deparaffinized, rehydrated, and loaded with 20 μg/mL Proteinase K for 30 min at the appropriate temperature. Then the slides were washed with PBS three times and incubated with TUNEL detection solution for 1 h in the dark. The green fluorescence of apoptotic cells was captured by a fluorescent microscope. Three images were evaluated.

### Microarray

To identify genes regulated by LINC-PINT, LINC-PINT-overexpressing HNE1 cells and control group cells were selected for microarray analysis. In Brief, total cellular RNA was extracted using the RNAiso Reagent (Takara, Japan), and qualified RNA was then purified by mirVana miRNA Isolation Kit (Ambion, Austin, TX, USA). After purification, cDNA was amplified using a poly dT-T7 promoter primer and covalently coupled with Cyanin3 (Cy3). Labeled cDNA samples were then subjected to SurePrint G3 Human Gene Expression 8x60K v2 Microarray Kit (Agilent). For downstream analysis, gene array data were analyzed and filtered using the GeneSpring software V13 (Agilent) according to the manufacturer’s instructions. Relative to control, signal intensity >onefold change and *P* < 0.05 were considered as differentially expressed genes, which were induced by LINC-PINT overexpression. For functional gene enrichment, GO analysis was performed and the *P* value was calculated by Bonferroni corrected Fisher’s exact tests.

### RNA pull-down assay

RNA pull-down assay was carried out by the BersinBio^TM^ RNA pulldown Kit (Cat no: Bes5102, BersinBio, CHN) according to instructions. In brief, LINC-PINT RNAs were transcribed and labeled by the Biotin in vitro. To obtain an appropriate secondary structure formation, biotinylated LINC-PINT was pretreated with RNA structure buffer and RNase-free water at room temperature. Subsequently, biotinylated RNA was added to streptavidin magnetic beads, followed by incubation with whole-cell lysate of NPC at 25 °C for 2 h. The proteins were retrieved from the protein-bead-RNA mixture by rinsing with protein elution buffer. SDS-PAGE and silver staining were used to separate and visualize specific bands. Finally, mass spectrometry (MS) was performed to identify specific proteins.

### Ribonucleoprotein immunoprecipitation (RIP)

For the RIP experiment, RNA Immunoprecipitation Kit (Cat no.: Bes5101, BersinBio, CHN) was used to validate the binding relationship between LINC-PINT and DNA-PKcs protein. To move DNA, NPC cells at around 80% confluency were scraped off and then treated with polysome lysis buffer, which was supplemented with DNase salt stock. The lysate was then incubated with anti-DNA-PKcs antibody or immunoglobulin G (IgG) control overnight at 4 °C, followed by treatment with protein A/G magnetic beads. To purify RNA, the beads were incubated with proteinase K at 55 °C after washing. Finally, purified RNA was isolated using phenol–chloroform extraction, and the relative enrichment of LINC-PINT was analyzed by qRT-PCR.

### Immunofluorescence

For immunofluorescence, NPC cells transfected with plasmids were seeded on a confocal dish (Cat no.: 801002, NEST, CHN), exposing to 8 Gy of irradiation when cells were grown to ~70% confluency. The cells were harvested at 0, 1, 2, and 4 h and then fixed, permeabilized, and blocked by using the Image-iT^TM^ Fixation/ Permeabilization Kit (Invitrogen, Carlsbad, CA, USA). Blocked cells were incubated overnight with the appropriate primary antibody at 4 °C. After washing in cold PBS three times, Invitrogen Alexa Fluor Plus 594 goat anti-rabbit IgG secondary antibody or Alexa Fluor Plus 488 goat anti-mouse IgG secondary antibody were incubated. Subsequently, cells were washed and sealed with an Invitrogen ProLong™ glass antifade mounting medium. NucBlue™ Stain was used to counterstain nuclei. All immunofluorescence images were obtained and visualized by LSM 900 (Zeiss, Jena, Germany) confocal microscope using a 63×oil immersion objective lens. Results were analyzed by Image J (Media Cybernetics, Inc., Rockville, MD, USA).

### Statistical analyses

Statistical analysis was performed using GraphPad Prism 5.0 software and SPSS 19.0 (SPSS Inc, Chicago, IL)). The Student’s *t* test (unpaired, two-tailed) was calculated to compare the differences between the two groups. We used Spearman rank correlation to analyze correlations between LINC-PINT and other genes. All data were confirmed in triplicate. Values in all graphs are represented as mean ± SEM, and *P* values <0.05 were considered significant. Significance is performed by an asterisk (*P < 0.05, **P < 0.01, ***P < 0.001 vs. the control).

## Supplementary information

Supplementary Figure 1

Supplementary Table S1. The clinicopathological parameters of nasopharyngeal carcinoma patients (n=90).

Supplementary Table S2. The information of primary antibodies.

## Data Availability

The datasets used and/or analyzed during the current study are available from the corresponding author on reasonable request.
