# Supplementary figures and images for: LINC-PINT impedes DNA repair and enhances radiotherapeutic response by targeting DNA-PKcs in nasopharyngeal cancer
Source: Cell Death Dis. 2021 May 7;12(5):454. doi: 10.1038/s41419-021-03728-2 (PMC8105365; doi:10.1038/s41419-021-03728-2)

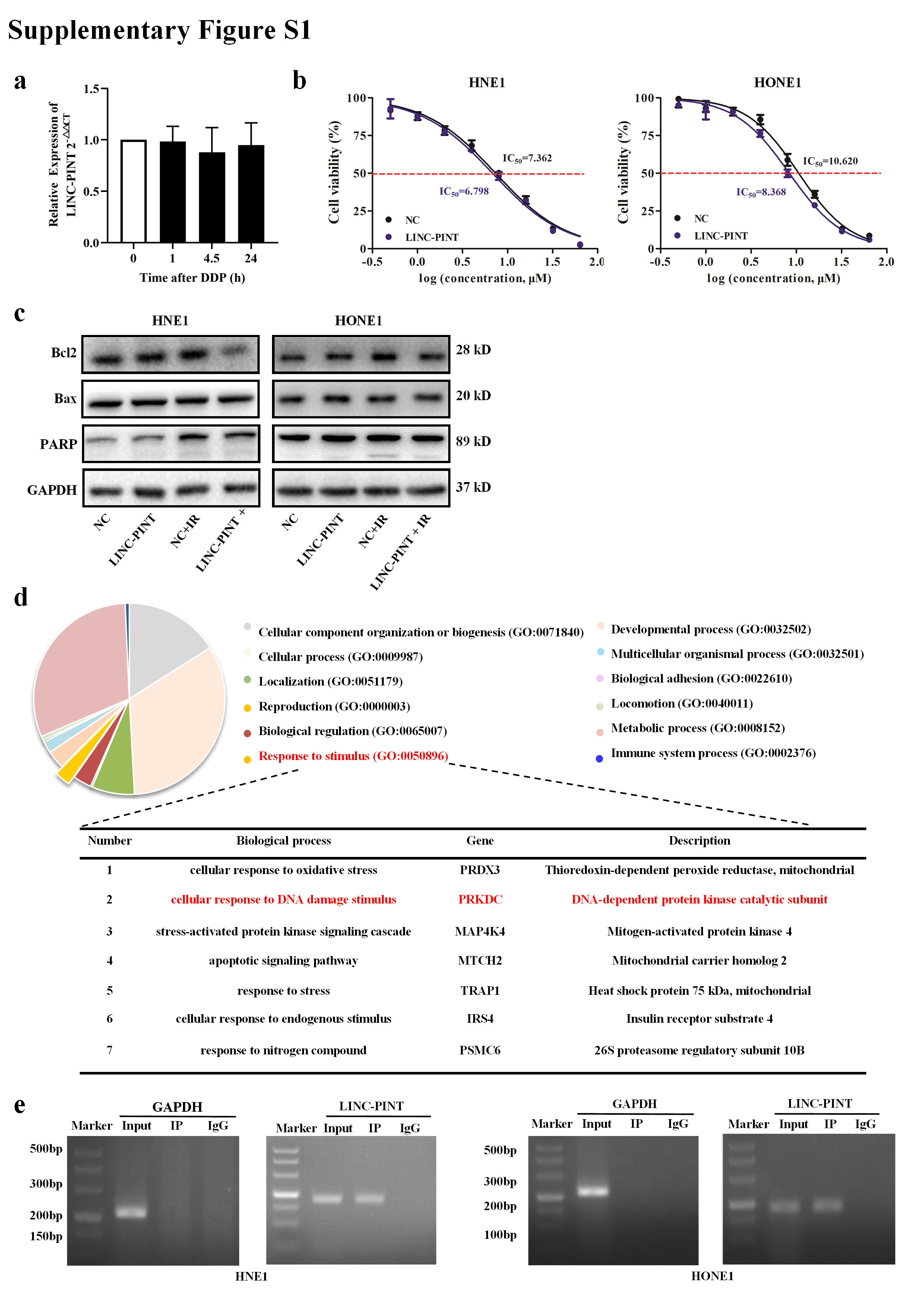

Supplement: Supplementary file 1 — Supplementary Figure 1 [file 41419_2021_3728_MOESM1_ESM.tif]
