# Supplementary material for: LINC-PINT impedes DNA repair and enhances radiotherapeutic response by targeting DNA-PKcs in nasopharyngeal cancer
Source: Cell Death Dis. 2021 May 7;12(5):454. doi: 10.1038/s41419-021-03728-2 (PMC8105365; doi:10.1038/s41419-021-03728-2)
Supplement: Supplementary file 2 — Supplementary Table S1. The clinicopathological parameters of nasopharyngeal carcinoma patients (n=90). [file 41419_2021_3728_MOESM2_ESM.docx]

**Supplementary Table S1. The clinicopathological parameters of nasopharyngeal carcinoma patients (n=90).**

| **Variable** | **No.** | **%** |
| --- | --- | --- |
| **Gender**  Male | 53 | 66.3 |
| Female | 27 | 33.7 |
| **Age** |  |  |
| ≥50 | 37 | 46.3 |
| <50 | 43 | 53.7 |
| **Primary tumor(T) stage** |  |  |
| T1-2 | 40 | 54.1 |
| T3-4 | 34 | 45.9 |
| **Lymph node(N) metastasis** |  |  |
| N0-1 | 12 | 16.2 |
| N2-3 | 62 | 83.8 |
| **Clinical stage** |  |  |
| I-II | 4 | 5.3 |
| III | 33 | 44.0 |
| IVa | 38 | 50.7 |
| **Radiotherapeutic response***  CR | 21 | 39.6 |
| PR | 23 | 43.4 |
| PD/SD | 9 | 17.0 |

* CR = complete response, PR = partial response, SD = stable disease and PD = progressive disease.
