# Supplementary material for: LINC-PINT impedes DNA repair and enhances radiotherapeutic response by targeting DNA-PKcs in nasopharyngeal cancer
Source: Cell Death Dis. 2021 May 7;12(5):454. doi: 10.1038/s41419-021-03728-2 (PMC8105365; doi:10.1038/s41419-021-03728-2)
Supplement: Supplementary file 3 — Supplementary Table S2. The information of primary antibodies. [file 41419_2021_3728_MOESM3_ESM.docx]

**Supplementary Table S2. The information of primary antibodies.**

| **Name** | **Cat No** | **Company** |
| --- | --- | --- |
| anti-GAPDH antibody | 2118 | CST |
| anti-ATM antibody | 2873 | CST |
| anti-phospho-ATM (Ser 1981) antibody | 5883 | CST |
| anti- phosphor-ATR (Ser428) antibody | 2853 | CST |
| anti-Rad50 antibody | 3427 | CST |
| anti-MRE11 antibody | 4847 | CST |
| anti-AKT antibody | 9272 | CST |
| anti-phospho-CHK1(Ser345) antibody | 2348 | CST |
| anti-phospho-CHK2(Thr68) antibody | 2661 | CST |
| anti-PARP antibody | 9532 | CST |
| anti-Bcl-2 antibody | 15071 | CST |
| anti-Bax antibody | 5023 | CST |
| anti-KU80 antibody | 16389-1-AP | Proteintech |
| anti-KU70 antibody | 10723-1-AP | Proteintech |
| anti-ATR antibody | 19787-1-AP | Proteintech |
| anti-NBS1 antibody | 55025-1-AP | Proteintech |
| anti-DNA-PKcs | ab70250 | abcam |
| anti-DNA-PKcs antibody | ab32566 | abcam |
| anti-DNA-PKcs (Ser2056) antibody | ab18192 | abcam |
| anti-Ku80 antibody | ab119935 | abcam |
| anti-Histone H2AX-S139 antibody | AP0099 | ABclonal |
